# The role of *in vitro* testing in pharmacovigilance for ß-lactam-induced serum sickness-like reaction: A pilot study

**DOI:** 10.3389/fphar.2022.945545

**Published:** 2022-08-30

**Authors:** Abdelbaset A. Elzagallaai, Awatif M. Abuzgaia, Blanca R. Del Pozzo-Magaña, Eman Loubani, Michael J. Rieder

**Affiliations:** ^1^ Departments of Paediatrics, Schulich School of Medicine and Dentistry, University of Western Ontario, London, ON, Canada; ^2^ Physiology and Pharmacology, Schulich School of Medicine and Dentistry, University of Western Ontario, London, ON, Canada

**Keywords:** drug hypersensitivity, pharmacovigilance, beta-lactam agents, serum sickness-like reaction, adverse drug reaction

## Abstract

**Background:** Current pharmacovigilance (PV) methods for detection of adverse drug reactions (ADRs) fail to capture rare immune-mediated drug hypersensitivity reactions (DHRs) due to their scarcity and the lack of clear diagnostic criteria. Drug-induced serum sickness-like reactions (SSLRs) are rare type of DHRs that occur in susceptible patients 1–3 weeks after exposure to the culprit drug with ß-lactam antibiotics being the most associated drugs. The diagnosis of drug induced SSLR is difficult due to the lack of safe and reliable diagnostic tests for identifying the culprit drug. The lymphocyte toxicity assay (LTA) is an *in vitro* test used as a diagnostic tool for drug hypersensitivity reactions (DHRs).

**Objective:** To evaluate the role of the LTA test for diagnosing and capturing SSLR due to ß-lactam antibiotics in a cohort of patients.

**Methods:** Patients were recruited from patients referred to the Drug Hypersensitivity Clinic at Clinic at London Health Science Centre with suspicion of drug allergy. Twenty patients (10 males and 10 females) were selected to be tested to confirm diagnosis. Demographic data was collected form the patents and blood samples were withdrawn from all patients and from 20 healthy controls. The LTA test was performed on all subjects and data is expressed as percentage increase in cell death compared to control (vehicle without the drug).

**Results:** In the result of LTA tests performed on samples from the selected 20 patients. There was a significant (*p* < 0.05) concentration-dependent increase in cell death in cells isolated from patients as compared to cells from healthy controls when incubated with the drug in the presence of phenobarbitone-induced rat liver microsomes.

**Conclusion:** Giving its safety and good predictive value the LTA test has very strong potential to be a useful diagnostic tool for ß-lactam-induced SSLR. The test procedure is relatively simple and not overly costly. Further studies including other drug classes are needed to evaluate the utility of the LTA test for SSLR due to other drugs.

## Introduction

Pharmacovigilance (PV) is defined by the World Health Organization (WHO) as the science and activities relating to detection, assessment, understanding and prevention of adverse effect or any other medicine-related problem (WHO, 2014). The importance of the discipline of PV is generally considered to have been established by the release of the Kefauver-Harris Amendment (Drug Efficacy Amendment) to the Federal Food and Cosmetic Act in the United States in 1962. The law required drug manufacturer to provide proof of effectiveness and safety of their drugs before approval (Peltzman, 1973). The terms “pharmacovigilance” and “drug safety” are commonly used in the field to describe the systematic collection and review of post-marketing drug safety data to guide drug use (Beninger, 2018). However, PV activities also include reviewing reports submitted by clinical investigators early during the drug development process and during selection of first safe human dose.

Adverse drug reactions (ADRs) are one of the leading causes of death in the developed world and represent a heavy cost burden on the healthcare system causing many hospital admissions and extended hospitalizations (Bates et al., 1997). ADRs cause one death every 5 min and cost over $136 billion annually in the United States (Johnson and Bootman, 1995). In the European Union, ADRs are estimated to be responsible for 5% of hospital admissions and cases 197,000 deaths annually (Bouvy et al., 2015). ADRs can either be type A, which are predictable from the drug pharmacology and dose dependent and type B, which are unpredictable, unrelated to the drug’s known pharmacology and do not have clear dose dependency. Type B ADRs include immune-mediated drug hypersensitivity reactions (DHRs; drug allergy) and non-immune mediated DHRs (also called pseudoallergy). They represent smaller fraction of total ADRs (−15%–20%) with some types of reactions lie under the rare and very rare categories (i.e., incidence between ≥1/10,000 to 1,000 and <1/10,000 of drug exposure, respectively). Rare and very rare ADRs cannot be captured during the pre-marketing stages of drug development due to the underpowered sample size (Chan et al., 2015). In addition, it is always not feasible nor practical to study unpredictable (type B) ADRs in prospective, interventional, and clinical trial studies due to their unpredictability and rare occurrence. Another inherited problem associated with these reactions is the difficulty in defining cases based on clinical presentation and associated signs and symptoms (Uetrecht and Naisbitt, 2013). Many of these rare ADRs are underreported due to poor case definition and lack of diagnostic methods (Lopez-Gonzalez et al., 2009). In fact, it is estimated that over 95% of ADRs go unreported (Bailey et al., 2016). This is a major problem as the only way to fully evaluate drug safety in real world is though robust pharmacovigilance studies and data collection. Many drugs have met all the regulatory efficacy and safety requirements only to be later withdrawn from the market due to efficacy or safety concerns jeopardizing patient safety and costing the drug developers and the healthcare systems billions of dollars (Qureshi et al., 2011). It is therefore extremely important to develop sensitive and specific methods to detect and report ADRs in the early stages of clinical use. The current PV systems, which largely depends on spontaneous voluntarily reporting lack such robustness and fundamentally inefficient to detect signal from noise due to lack of reliable diagnostic test to identify cases (Salvador et al., 2022). We propose that a reliable *in vitro* diagnostic test for rare and very rare idiosyncratic ADRs would help capture and report them enhancing PV and ADR surveillance. Efficiency of surveillance is particularly essential for rare and very rare ADRs; for instance, missing one case of an ADRs with 5% prevalence may not have a significant effect on the overall surveillance process but missing one case 1 in 10,000 exposures may result in failure to detect the ADR leading to unsafe exposure of a large number of patients to the drug.

DHRs are divided, according to the immune mechanism and type of immune cells involved, into type I (IgE-mediated), type II (cytotoxic reactions mediated by drug-specific IgG), type III (immune complex-mediated), and type IV reactions (delayed reactions, T-cell-mediated) (Elzagallaai and Rieder, 2015). Serum sickness (SS), which belongs to type III immune-complex mediated reaction, was first described by von Pirquet and Schick in 1951 (von Pirqet and Schick, 1951). It was later found that circulating immune complexes and complement activation is important in the pathophysiology of these immune-mediated reactions (Vaughan et al., 1967). Serum sickness-like reaction (SSLR) is clinically similar reaction that mostly triggered by drugs. They are most associated with ß-lactam antibiotics (especially cefaclor and amoxicillin), sulfonamides, fluoroquinolones, aromatic anticonvulsants, tetracyclines, minocycline, metronidazole, bupropion, and other drugs including biologicals (Lawley et al., 1984; Platt et al., 1988; Heckbert et al., 1990; Weiss and Smith, 2020). This type of reactions can also develop as a result of vaccine administration including recent cases of SSLR to inactivated COVID-19 vaccine (Chung et al., 2021; Chaijaras et al., 2022). The condition is defined by sudden appearance of skin rash (usually urticaria-like) and arthritis usually manifested 1–3 weeks after drug exposure, which can be accompanied by fever, lymphadenopathy, eosinophilia, and rarely renal involvement (Del Pozzo-Magana et al., 2021). It is uncommonly seen in clinical practice, but its incidence appears to be on the rise since the introduction of biologic drugs (Finger and Scheinberg, 2007; Khan, 2016). It has been estimated that the incidence of SSLR associated with cefaclor is between 0.024% and 0.2% per course (Knowles et al., 2000). The diagnosis of SSLR is challenging due to other possible causes (Schryver, 2015). The exact prevalence of SSLR due to ß-lactam antibiotics is not known, however, studies have estimated it to complicate 0.4%–0.5% of antibiotic courses (Reynolds, 1996; Isaacs, 2001). In a 10-year retrospective cohort study we found that SSLR represent 15.4% of all patients with cutaneous ADRs referred to our clinic, 0.02% of all cause of consult, and 0.9% of all sudden skin rashes seen in the our institution pediatric emergency department. The most commonly implicated drugs were ß-lactam antibiotics including amoxicillin (87%) and cephalosporins (8.5%) (Del Pozzo-Magana et al., 2021). Type B ADRs also include “pseudoallergy”, which is non-immune-mediated. Examples of the latter reactions are opioids-induced pruritis and NSAIDs-induced pseudoallergy (Zhang et al., 2018). Another example of pseudoallgy is complement activation-related pseudoallergy (CARPA) (Szebeni, 2005).

The precise details of the pathophysiology of drug-induced SSLR is not well understood. However, in delayed onset drug hypersensitivity the generation of cytotoxic reactive metabolites from drug molecules *in vivo* is believed to be the first step in a cascade of events leading to the immune-mediated reaction (Elzagallaai et al., 2017). These reactive metabolites are capable of adducting (hapenating) endogenous macromolecules produced an antigen recognized by the immune system as non-self. They may also cause local or systemic cell damage resulting in releasing ‘danger signals’, which prime immune cells to mount the reaction (Matzinger, 1994; Pichler et al., 2010x). Diagnosis of drug-induced SSLR is challenging, mainly based on clinical presentation and medication history, and no reliable and safe diagnostic test is available. Case definition for management and pharmacovigilance purposes is therefore challenging giving the fact that presenting signs and symptoms are often variable.

The lymphocyte toxicity assay (LTA) is an *in vitro* test that has been proven to have a significant value in the diagnosis of drug-induced hypersensitivity reactions (DHRs) but most of the validation work has been focused on type IV T-cell-mediated delayed DHRs (Elzagallaai et al., 2013; Dhir et al., 2021). The test has been shown in a study involving 51 patients with DHRs to have a sensitivity of 99% and specificity of 75% (Naranjo et al., 1994). In another study, Neuman et al. used the test to investigate DHRs in 86 patients with suspected reaction to sulfamethoxazole and 62 patients with suspected reactions to anticonvulsants (Neuman et al., 2000). They estimated the test sensitivity and specificity to be 98% and 89%, respectively. Other studies have estimated the positive predictive value of the LTA in cases of DHRs to sulfonamides to be between 80% and 90% (Neuman et al., 2002; Neuman et al., 2007). Using re-exposure as a gold standard test in a small cohort (22 patients), we calculated the overall sensitivity and specificity of the LTA test to be 40% and 90%, respectively, but that depended on the suspected drug (Elzagallaai et al., 2010). In this study we explored the potential role of the LTA *in vitro* test for diagnosis of ß-lactam-induced SSLRs for the purpose of optimizing and improving pharmacovigilance to these rare types of DHRs.

## Materials and methods

### Materials

Penicillin, cephalexin, tetrazolium salt 3-(4, 5-dimethylthiazol-2-yl) 2, 5 diphenyl-tetrazolium bromide (MTT), hydrogen peroxide (H_2_O_2_), 2′,7′-dichlorofluorescin diacetate (DCFH-DA), Histopaque® -1077 (Ficoll), Hank’s balanced salt solution (HBSS) and dimethyl sulfoxide (DMSO) were purchased from Sigma-Aldrich (St, Louis, MO, United States). RPMI 1640 and trypan blue were purchased from Invitrogen™, Life Technologies Inc. (Burlington, ON, Canada). Phenobarbitone-induced pooled male Sprague-Dawley rat liver microsomes were purchased from BioIVT (Westbury, NY, United States). All other chemicals used in this study were the highest purity commercially available.

### Subjects

Informed consent was obtained from all participants and the study protocol was approved by the Western University Research Ethics Board for Human Subjects (REB No. 11883E). Two groups of individuals were included in our study. The first group consisted of patients who had experienced a β-lactam antibiotic-induced SSLR. The diagnosis of SSLR was established by revising patients’ files by two clinicians (AMA and BD-M), who have experience in managing patients with DHRs. Any ambiguity in the diagnosis was confirmed by a third clinician (MJR). The general criteria for diagnosis include development of skin rash and joint inflammation with or without fever after exposure to the culprit drug (De Schryver and Ben-Shoshan, 2015; Del Pozzo-Magana et al., 2021). The inclusion criteria of this group include: 1) Having a history of SSLR related to the administration of a ß-lactam antibiotics (penicillins or cephalosporins); 2) symptoms developed are highly suggestive of SSLR and should include skin rash and joints involvements; 3) the patient consents to participate in the study and provides a sufficient blood sample. We excluded patients with any underlying rheumatological conditions (e.g., lupus, rheumatoid arthritis, dermatomyositis, spondyloarthropathies, Sjogren’s disease. Juvenile idiopathic arthritis, and polymyalgia rheumatica). The second group is composed of 20 healthy individuals, who denied any history of DHRs to ß-lactam antibiotics. Overall, 20 patients between the age of 11 months and 67 years were recruited. The patients’ characteristics are summarized in Table 1.

**TABLE 1:** Characteristics of patients included in the study.

Patient #	Sex	Age (Y, years; M, months)	Drug involved	Onset of reaction (Days)	Type of skin rash	Presence of fever	Other symptoms	Time to resolution	Treatment
1	F	18M	Amox	7	MP	Y	JP&S	4	St
2	M	3Y	Amox	10	MP	N	JP&S	6	St
3	F	2Y	Amox	7	MP	Y	JP&S	5	St
4	M	32Y	Amox	10	MP	NA	JP&S	6	St
5	F	30M	Amox	3	UM	Y	JP	5	St
6	M	2Y	Amox	6	MP	N	JP&S	5	NA
7	M	29M	Amox	7	MP	N	JP	5	St
8	F	5.5Y	Amox	7	MP	Y	JP	5	St
9	F	19M	Amox	7	UM	NA	JP	5	AH
10	M	67Y	Ceph	NA	MP	NA	NA	NA	NA
11	F	3Y	Amox	7	EM	NA	JP	15	St
12	M	50Y	Amox	NA	MP	NA	JP&S	14	St
13	F	2Y	Amox	10	EM	Y	JP	6	St
14	M	6Y	Amox	10	MP	N	JP&S	14	St
15	M	8Y	Ceph	7	MP	y	Jp	5	St
16	M	2Y	Amox	7	EM	NA	JP&S	5	AH
17	F	2Y	Amox	5	MP	NA	JP&S	&	AH
18	M	11M	Amox	7	MP	NA	JP&S	5	AH
19	F	3Y	Amox	7	MP	Y	JP&S	3	AH
20	F	2Y	Ceph	8	MP	Y	JP&S	4	St

### Blood collection and isolation of cells

Thirty milliliters of peripheral venous blood samples were collected from each participant by venipuncture into heparinized syringes and processed immediately. To isolate peripheral blood monocytes (PBMCs), blood was diluted 1:1 with phosphate-buffered saline (PBS, 10 mM NaH_2_PO_4_, 2 mM KH_2_PO_4_, 137 mM NaCl, 2.7 mM KCl; pH 7.2) and 30 ml were layered over 15 ml of Ficoll-Paque density gradient and centrifuged at 500 g for 20 min. The interface layer (buffy coat) was then collected. Cells were washed twice with PBS and adjusted to 1×10^6^ cell/mL in HEPES [4-(2-hydroxyethyl)-1-piperazine] ethanesulfonic acid buffered saline containing 15 mM HEPES, 125 mM NaCl, 6 mM KCl, 1.2 mM MgSO_4_, 1.0 mM NaHCO_3_, 1.0 mM CaCl_2_, 10 mM glucose; pH 7.4).

### *In vitro* toxicity testing

The LTA was performed as described previously (Elzagallaai et al., 2010; Elzagallaai et al., 2011). Briefly, PBMCs were plated in flat-bottom 96-multi-well plates at a density of 1×10^5^ cells per well in quadruplicate and treated with a final concentration of 6.25–125 μg/ml of either amoxicillin or cephalexin depending on the suspected drug. Drug solutions were freshly prepared in dimethyl sulfoxide (DMSO) and diluted in culture media to give the desired final concentration (DMSO final concentration is always kept at ≤1%). Microsomal protein was added at a concentration of 0.25 mg/ml, followed by addition of the NADPH-generating system (nicotinamide adenosine dinucleotide phosphate [NADP] 0.6 mM, glucose-6-phosphate 2.4 mM, glucose-6-phosphate dehydrogenase 2 U/ml). Preparations were incubated for 2 h at 37°C in a 5% CO_2_ humidified atmosphere. A standard curve for measuring cell death was generated by seeding cells at 25%, 50%, 75% or 100% of cell populations in culture media in quadruplicate. After incubation, drugs in solution were removed by centrifugation at 500 *g* for 10 min. Then, cells were suspended in 100 μl fresh RPMI-1640 media supplemented with 10% FBS, 100U/ml penicillin G sodium and 100 μg/ml streptomycin sulfate, and left to recover for 18 h in an atmosphere of 5% CO_2_ at 37°C. Cell viability was quantified using MTT staining as described previously. (Elzagallaai et al., 2010).

### Statistical analysis

Statistical analysis was performed using Prism GraphPad software. The numbers of dead cells were expressed as a percentage of control (vehicle without drug) and blotted as mean ± standard error (SEM). Significant differences were determined by two-tailed Student’s t-test. A probability of more than 95% (*p* ≤ 0.05) was considered significant. Correlations were made using Pearson correlation analyses. Unless otherwise indicated, values are presented as mean ± standard error (SEM).

## Results

**FIGURE 1:**
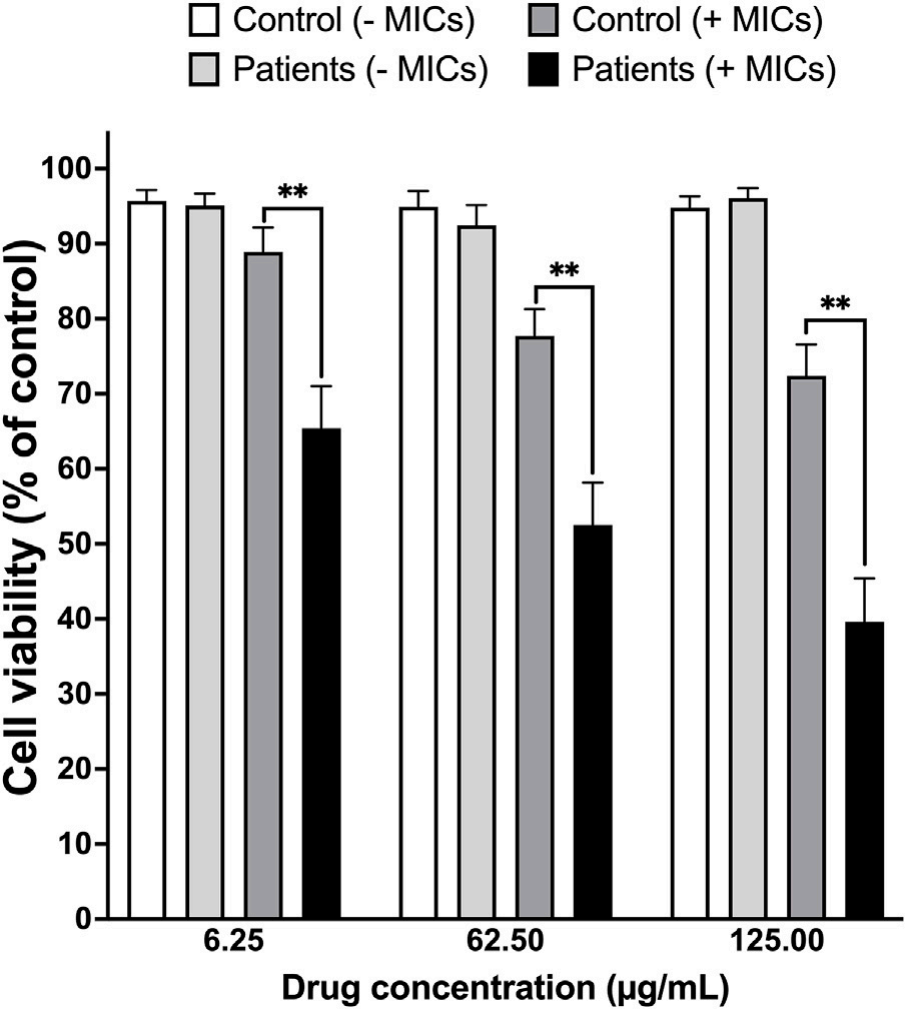
Summary of the LTA test results for 20 SSLR patients and 20 healthy controls. Lymphocytes isolated from health controls were incubated with drug either without microsomes (MICs, white bars) or with drug and MICs (light grey bars) and cells isolated from SSLR patients were incubated with drug either without MICs (dark grey bars) or with MICs (black bars). Y axis represents cell viability expressed as a percentage from incubating the corresponding cells with control vehicle without the drug. **, *p* < 0.001.

**FIGURE 2:**
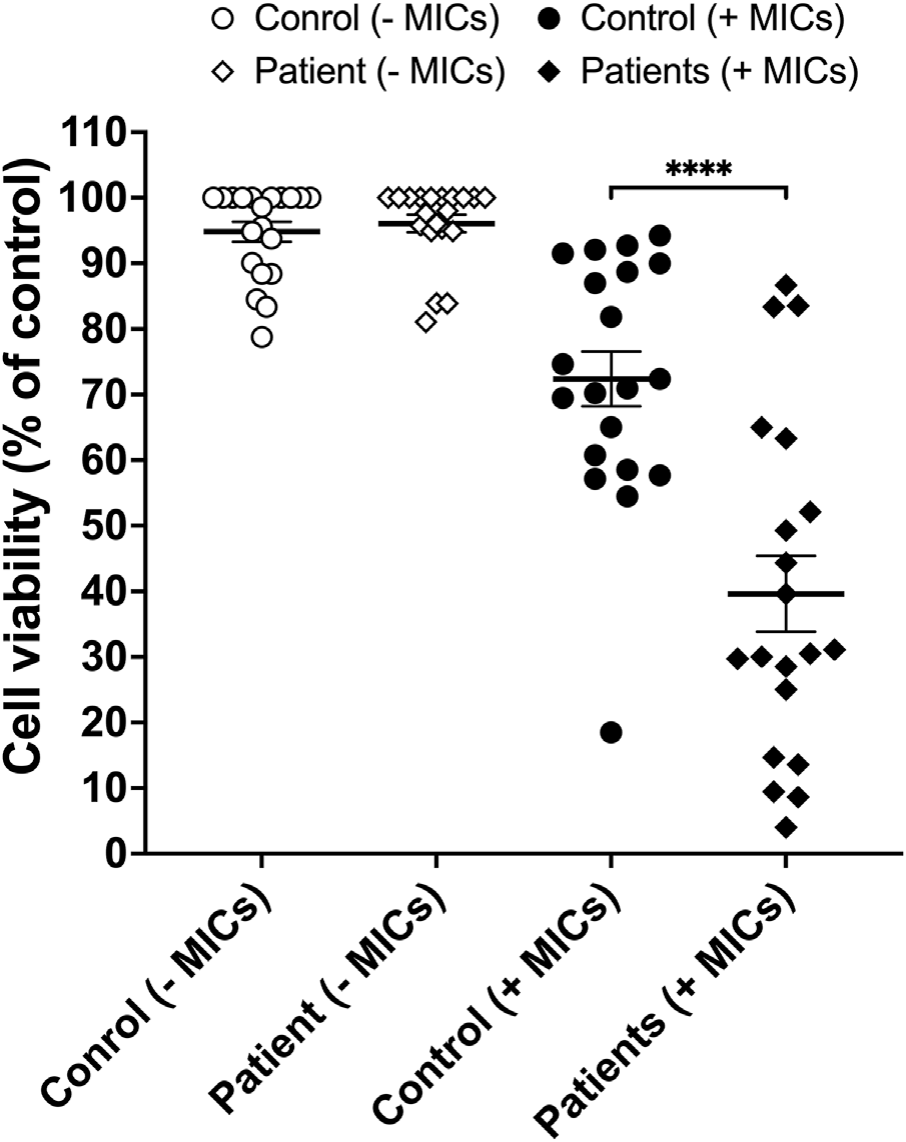
Cell viability (expressed as % of control) of PBMCs from healthy volunteers without microsomes (MICs, open circles), SSLR patients without MICs (open diamonds), healthy controls with MICs (closed circles) and SSLR patients with MICs (closed diamonds). Mean ± SEM. ****, *p* < 0.0001.

Twenty patients (10 males and 10 females) presented with symptoms that meet our inclusion criteria for SSLR to beta-lactam antibiotics (penicillins and cephalosporins). Clinical symptoms included cutaneous lesions (maculopapular, EM, urticaria) and joint inflammation (arthritis) that included hands, feet, or both. Eight of the 20 patients also developed fever as part of the hypersensitivity syndrome. The mean age of the patients was 9.9 years and ranged from 11 months to 67 years. The characteristics of the patient population is summarized in Table 1. All patients had positive LTA test results using a cut-off value of 20% increase in cell death (Figure 1). At 125 μM of the drug and in the presence of MICs, degree of cell death was significantly higher (*p* < 0.0001) in cells isolated from ß-lactam-induced SSLR patients (Mean: 60.37%) than cells from healthy controls (Mean: 27.61%). Difference between means (controls and patients) ± SEM = 32.76 ± 7.117 (95% confidence interval: 47.17 to 18.35) (Figure 2).

## Discussion

Case definition in PV studies require applying rigorous criteria, which in cases of idiosyncratic reactions are almost always lacking. In addition, use of PV algorithms in the diagnosis of DHRs in general is not accurate because of the often lack of sufficient information for scoring (Benahmed et al., 2005). An alternative would be a reliable and safe *in vitro* test with adequate sensitivity and specificity to detect true cases among the suspected cohort of patients. It is understandable that these criteria can only be applied in prospective PV studies but can be used for PV surveillances.

Drug-induced SSLR represent a major problem to healthcare—along with other idiosyncratic hypersensitivity reactions—due to the difficulty in diagnosis and accurate identification of the culprit drug. Approximately 10% of the general population report an allergy to β-lactam antibiotics; however, 90% of reported allergies to β-lactam antibiotic cannot be ruled out immunologically (Surtees et al., 1991). Such false labeling of patients puts them at greater risk of adverse reaction due to the use of less safe alternative drugs with inferior effectiveness to treat their infection which increases length of hospital stay and worsen the outcome. Furthermore, false labeling result in the use to non-beta-lactam antibiotics leading to cost increases and contributes to worsening the bacterial resistance problem. Capturing true cases of ß-lactam-induced SSLR using available clinical criteria is difficult due to lack of reliable diagnostic tests. On the other hand, for newly marketed drugs, especially biologicals, capturing IDRs for safety evaluation is utmost important for proper PV monitoring. All the available diagnostic aids including skin testing and oral re-challenge have their risks and shortcomings and are not always feasible to perform either due to lack of expertise or fear of inducing a severe reaction in the patient. The LTA has the advantage of being safe as an *in vitro* test and can be used both as a diagnostic test and an investigative tool for the pathophysiology of SSLRs. Kearns et al. (Kearns et al., 1994) tested 19 patients (10 male and 9 females) suspected of developing SSLR to cefaclor and found that subjects with SSLR exhibited an increase in cell death of 50%–167% above baseline. The effect was specific to cefaclor and was not produced by incubation of isolated cells with another cephalosporin (cephalexin) along with metabolic activation system (Kearns et al., 1994). In another study, the same group also tested 10 patients with SSLR to cefaclor using the LTA test. The degree of cell death in the patient pollution was highly positive and ranged from 40% to 140% increase above baseline (Kearns et al., 1998). In a validation study for the LTA test using systemic re-exposure as a gold standard to determine the predictive value of the test for diagnosis of hypersensitivity reactions (HSRs) to different groups of drugs, we tested 11 patients with HSRs to beta-lactam antibiotics (6 to amoxicillin and 5 to cefaclor) (Elzagallaai et al., 2010). When the results of the re-exposure were compared to the LTA results, all except one patient had complete agreement.

The main pitfall associated with evaluating the role of *in vitro* toxicity testing for pharmacovigilance monitoring of rare drug-induced reactions is the lack of large studies looking at the predictive value of these tests (Elzagallaai et al., 2009). We speculate that one of the main reasons for this is the technical skills and special equipment required to perform the test restricting it to highly sophisticated research centers. We have introduced a more simplified version of the LTA test—the *in vitro* platelet toxicity assay (iPTA)—using blood platelets as a surrogate cell model for toxicity testing (Elzagallaai et al., 2011). The iPTA test has been proven to be less technically demanding and less expensive than the LTA with potentially better predictive value (Elzagallaai et al., 2013).

Data from this pilot study points to the value of the LTA (and potentially the iPTA) both as a diagnostic tool for beta-lactam-induced SSLRs and as a PV monitoring tool. Further research with larger numbers of patients is needed to further explore the pathophysiology and biology of SSLR to β-lactam antibiotics.

## Data Availability

The raw data supporting the conclusions of this article will be made available by the authors, without undue reservation.

## Abbreviations

AH, antihistamine; Amox, amoxicillin; Ceph, cephalexin; EM, Erythema multiforme; JP, Joint pain; JP&S, Joint pain and swelling; MP, Maculopapular; NA, not available; St, steroids; UM, Urticaria multiforme.
